# Supplementary material for: Quantitative trait loci (QTL) underlying phenotypic variation in bioethanol-related processes in Neurospora crassa
Source: PLoS One. 2020 Feb 4;15(2):e0221737. doi: 10.1371/journal.pone.0221737 (PMC6999864; doi:10.1371/journal.pone.0221737)
Supplement: S10 File — This file contains sequence information of 4-nucleotide deletion identified in FGSC4825. (PDF) [file pone.0221737.s010.pdf]

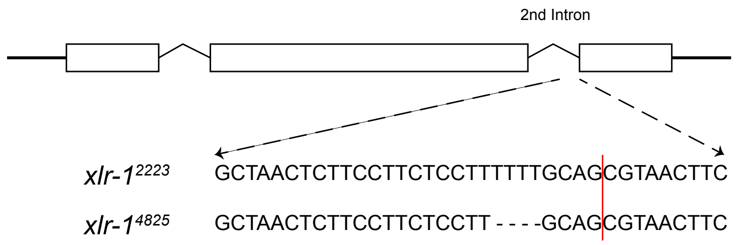

The deletion mutation identified in the second intron region. The red vertical line represents the splice acceptor site.
